# Prehospital tranexamic acid for trauma victims

**DOI:** 10.1186/s40560-023-00661-8

**Published:** 2023-03-22

**Authors:** Kazuhiko Omori, Ian Roberts

**Affiliations:** 1 Centre for Trauma Sciences, Blizard Institute, Queen Mary University of London, 4 Newark Street, London, E1 2AT UK; 2 London School of Hygiene and Tropical Medicine, London, WC1E 7HT UK

**Keywords:** Haemorrhage, Trauma, Tranexamic acid, Intramuscular, Prehospital care

## Abstract

The public enquiry into the mass casualty incident at the Manchester Arena in the UK in which 23 people died and over 1000 were injured, identified the need for timely intramuscular administration of tranexamic acid to trauma patients. Since then, a number of studies and trials have been carried out and UK paramedics are now authorized to give intramuscular tranexamic acid in the pre-hospital setting. In Japan, pre-hospital administration by emergency life-saving technicians is not yet authorized, despite the fact that tranexamic acid was invented by Japanese scientists. In Japan, the need for the pre-hospital administration of tranexamic acid has been raised on several occasions, where a patient died from traumatic bleeding prior to hospital admission. This paper summarizes the evidence on the use of tranexamic acid in patients with traumatic bleeding, including new evidence on the intramuscular route.

## Background

Injuries from accidents and violence are a leading cause of death in children, adolescents, and young people. Many of the deaths are from bleeding and traumatic brain injury. Although most injury deaths receive little or no media attention, injury deaths of high-profile people and mass casualty events are widely covered by the press and sometimes result in public inquiries that ask whether and how they could have been prevented or better managed.

On the 22nd May 2017, a suicide bomber detonated a shrapnel filled bomb just as people were leaving an Ariana Grande concert in the Manchester Arena in the UK. Twenty-three people were killed and over 1000 people were injured, many of them children. Sir John Saunders led the public inquiry (Manchester Arena Inquiry) that asked what the emergency services might have done to reduce the loss of life. In November 2022, he published his recommendations [1]. Aware that tranexamic acid is known to reduce mortality in bleeding trauma victims and was given to the victims of the Bataclan attack in Paris, the Inquiry investigated whether victims of the Manchester Arena bombing had received tranexamic acid. Noting the difficulties of giving intravenous tranexamic acid to a large number of casualties, Sir John asked whether intramuscular tranexamic acid administration might have saved lives:

“Intravenous administration of Tranexamic acid (TXA) may be difficult in patients lacking sufficient volume of blood. It takes approximately ten minutes to administer, during which period the paramedic must remain with the patient. That will cause delay in the treatment of other patients in a mass casualty situation. Both problems could be solved by the use of intramuscular as opposed to intravenous TXA.”

Sir John recommended that a review be carried out by the UK Department of Health and Social Care into whether ambulance crews should carry intramuscular TXA. This paper aims to inform the discussion by summarizing the evidence on the use of tranexamic acid in bleeding trauma victims including new evidence on the intramuscular route.

## Tranexamic acid

Tranexamic acid (TXA) was invented by Japanese scientists Shosuke and Utako Okamoto shortly after World War II [2]. TXA is an antifibrinolytic drug that reduces bleeding by inhibiting the enzymatic breakdown of fibrin blood clots. Plasminogen, a glycoprotein pro-enzyme produced in the liver, is converted into the fibrinolytic enzyme plasmin by tissue plasminogen activator (tPA). The plasminogen molecule is folder into loops called kringles that protrude like fingers. These fingers bind to fibrin via lysine-binding sites on their tips. If the lysine residues on fibrin are enzymatically removed, plasminogen binding is inhibited. Fibrin binds both plasminogen and tPA which localizes and promotes plasmin production. Plasmin that is bound to fibrin is shielded from plasmin inhibitors. Plasmin cuts fibrin into fibrin degradation products. This exposes more lysine molecules which bind more plasminogen, thus accelerating fibrinolysis. TXA has a molecular structure similar to lysine and inhibits fibrinolysis by inhibiting the binding of plasminogen to fibrin.

### Surgical bleeding

TXA is widely used to reduce bleeding and the need for blood transfusion in surgical procedures and is the subject of much recent research. A systematic review and meta-analysis in 2012 showed that TXA reduced the need for blood transfusion by a third (risk ratio 0.62, 95% confidence interval 0.58 to 0.65; *P* < 0. 001) in 129 trials conducted between 1972 and 2011, involving a total of 10,488 patients (risk ratio 0.62, 95% confidence interval 0.58 to 0.65; *P* < 0.001) [3]. A subsequent meta-analysis examining data from 104 clinical trials showed that TXA administered at the time of incision, regardless of the type of surgery or extent of bleeding shown to reduce bleeding by approximately one-third (pooled ratio 0·66, 95 per cent confidence interval 0·65 to 0·67; *P* < 0·001) [4].

In 2017, the Aspirin and TXA for Coronary Artery Surgery (ATACAS) trial, a large, high-quality, international randomized trial in 4662 patients undergoing cardiac surgery, showed that TXA reduced the risk of postoperative bleeding with a significant reduction in blood transfusion (relative risk, 0.69; 95% confidence interval [CI], 0.65–0.74; *P* < 0.001). There was no evidence of any increased risk of vascular occlusive events [386 (16.7%) deaths or thrombotic complications occurred within 30 days after surgery in the tranexamic acid group and 420 (18.1%) in the placebo group (relative risk, 0.92; 95% confidence interval [CI], 0.81 ~ 1.05; *P* = 0.22)] [5]. More recently, the Perioperative Ischemic Evaluation-3 (POISE-3) trial randomly assigned 9535 adults at risk of bleeding and cardiovascular complications undergoing non-cardiac surgery to tranexamic acid or matching placebo in 2022. There was no evidence of any increased risk of vascular occlusive events (occurring in 14.2% of patients in the tranexamic acid group and 13.9% in the placebo group (hazard risk [HR] = 1.02; 95% CI, 0.92–1.14)) and TXA reduced the risk of major bleeding by approximately 25% (hazard risk [HR] = 0.75; 95% CI, 0.65–0.87) [6, 7]. Widespread use of TXA would improve surgical safety, avoid unnecessary blood use, reduce the risk of infections from blood transfusions and save healthcare costs [8].

### Traumatic bleeding

In 2010, the Clinical Randomization of an Antifibrinolytic in Significant Haemorrhage (CRASH)-2 trial, the largest ever clinical trial of TXA in trauma [double-blind RCT (randomized controlled trial)] evaluating the efficacy of TXA in bleeding trauma patients, showed that TXA safely reduces death due to bleeding (489 [4.9%] tranexamic acid group vs 574 [5.7%] placebo group; relative risk 0.85, 95% CI 0.76–0.96; *p* = 0.0077) and all-cause mortality (1463 [14.5%] tranexamic acid group vs 1613 [16.0%] placebo group; relative risk 0.91, 95% CI 0.85–0.97; *p* = 0.0035) in trauma victims [9].

It showed that early treatment is essential. TXA should be given as soon as possible after injury. Beyond three hours of injury TXA given after 3 h seemed to increase the risk of death due to bleeding (144/3272 [4.4%] events in tranexamic acid group vs 103/3362 [3.1%] in placebo group; relative risk [RR] 1.44, 1. 12–1.84; *p* = 0.004) [10]. Following the results of the CRASH-2 trial, TXA was included in trauma treatment guidelines, but patients with isolated traumatic brain injury were excluded. The CRASH-3 trial, published in 2019, examined the efficacy of TXA in patients with isolated traumatic brain injury (TBI) and found that in patients treated within 3 h of injury, the risk of death related to head injury was 18.5% in the tranexamic acid group and 19.8% in the placebo group (855 vs 892 events, risk ratio [RR] 0.94 [95% CI 0.86–1.02]), suggesting that treatment within 3 h of injury reduces death related to head injury [11]. The treatment effect was largest in patients with mild and moderate TBI, most probably because there is greater potential to prevent bleeding in these patients. Patients with severe TBI often had major intracranial bleeding prior to randomization. The CRASH-4 trial is underway and will examine the risks and benefits of intramuscular TXA in older adults with mild TBI [12].

### Postpartum bleeding

TXA was invented in the hope that it might reduce mortality from postpartum bleeding. The World Maternal Antifibrinolytic (WOMAN) Trial was a large multi-centre randomized placebo-controlled trial of TXA treatment in women with postpartum haemorrhage. The results showed that TXA reduced the risk of haemorrhage death by one fifth (155 [1.5%] of 10,036 patients in the tranexamic acid group vs 191 [1.9%] of 9985 in the placebo group, risk ratio [RR] 0·81, 95% CI 0·65–1.00; *p* = 0.045) without any increase in adverse events. As with CRASH-2 trial, early administration was most effective (89 [1.2%] in the tranexamic acid group vs 127 [1.7%] in the placebo group, RR 0.69, 95% CI 0.52–0.91; *p* = 0.008) [13]. TXA is therefore recommended to be administered as soon as possible in cases of severe obstetric haemorrhage. Based on this finding, World Health Organization (WHO) strongly recommends early intravenous TXA administration (and only within 3 h of birth) in addition to standard treatment for women clinically diagnosed with postpartum haemorrhage after vaginal delivery or caesarean section in 2017 [14].

### The importance of time to treatment and the need for pre-hospital use

**Fig. 1:**
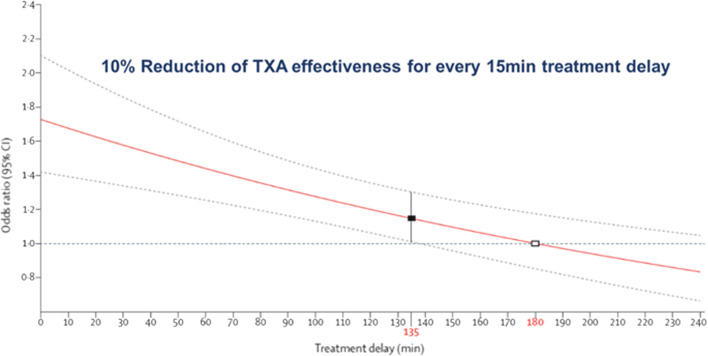
Effect of treatment delay on the survival benefit from tranexamic acid. The treatment effect is shown on the Y axis here and this is the time on the X axis. A big effect of tranexamic acid getting smaller with time until about 3 h there is no benefit at all. This figure is taken from reference [15] and copyright issues have been properly handled with the publisher

The importance of time to treatment was emphasized in an individual patient-level data meta-analysis (individual participant data) involving over 40,000 patients from the WOMAN and CRASH-2 trials. The study found that for every 15 min treatment delay there is a 10% reduction in the survival benefit from TXA treatment. The significant effect of TXA diminishes with time and after three hours it is no longer effective [15] (Fig. 1). Ideally, TXA should be administered in the first hour, preferably by paramedics at the scene of the injury. To reduce delays in treatment, TXA is increasingly administered by paramedics at the scene of injuries and in ambulances in the UK and USA. It takes a considerable amount of time for the ambulance to arrive, prepare to take the patient to hospital and to transport the patient to hospital. Data from trauma audits in the UK show that when paramedics provide pre-hospital treatment, the median time from injury to TXA treatment is 49 min (interquartile range 33–72). On the other hand, when given in hospital, the median time to treatment is 111 min (interquartile range 77–162) which is considerably less effective [16]. The current state of pre-hospital care in Japan is that in 2020, the average time required to admit a patient to the hospital (the time taken from receiving a 119 call to transferring the patient to a doctor) is approximately 41 min, with 10.1% of cases taking more than 60 min. In mountainous areas and areas where the number of emergency hospitals is decreasing due to a shortage of doctors, there is more concern about longer emergency transport times and the time taken to intervene medically [17]. This is why TXA needs to be administered by the emergency services in the pre-hospital phase.

The STAAMP trial published in 2020, was a randomized trial of pre-hospital TXA administration in 927 trauma patients in the USA. This was the first trial of tranexamic acid in US patients with polytrauma. One month following the injury, 8% of patients who received tranexamic acid had died compared with 10% in the placebo group, an approximate 20% reduction in mortality (8.1% in the tranexamic acid group vs 10.0% in the placebo group; risk ratio [RR] 0.82 [95% CI 0.60–1.11]). Although they observed a larger mortality reduction than seen in the CRASH-2 trial (about 15% reduction), the reduction was not statistically significant, most probably because of the small sample size. Although the findings of the STAAMP trial confirmed the results of CRASH-2, the STAAMP trial mistakenly concluded: “Prehospital administration of tranexamic acid compared with placebo did not result in a lower rate of 30-day mortality in this population.” However, no significant difference is not the same as no difference. The STAAMP trial was small but when combined in a meta-analysis of all large trials of tranexamic acid in trauma, the results only strengthen the conclusion that timely tranexamic acid administration is lifesaving [18, 19].

### Tranexamic acid can be given by intramuscularly injection

While early TXA in the pre-hospital setting is becoming more widespread, intravenous administration is a major obstacle to the timely administration of TXA worldwide. If TXA could be administered intramuscularly, ideally using an easy-to-use auto-injector, it could be administered by trained emergency life-saving technicians, non-medical personnel and other lay people, significantly reducing time to treatment. A systematic review of in vivo and in vitro pharmacodynamic studies was conducted to examine whether intramuscular injection of TXA is feasible, and found that TXA concentrations of 10–15 mg/l to inhibit fibrinolysis are suitable targets for pharmacokinetic studies, but TXA concentrations above 5 mg/l were also found to be potentially effective[20]. Subsequently, a randomized open-labelled crossover study of 1 g TXA intravenously, 1 g TXA intramuscularly and 2 g TXA orally was conducted, which confirmed that bioavailability was 1.0 for intramuscular and 0.47 for oral, with rapid and complete absorption of TXA intramuscularly [21]. In a recent prospective pharmacokinetic study conducted in the emergency departments of two large trauma centres in London, a loading dose of 1 g of TXA was administered intravenously according to guidelines in patients with traumatic bleeding and a second dose of TXA was given in two 5 ml (0.5 g each) intramuscular injections. As a result, a TXA concentration of 5 mg/l was reached in about 4 min after 1 g intramuscular administration and this level could be maintained for 10 h. If TXA was administered as a 0.5 g intramuscular injection, a TXA concentration of 5 mg/l would be achieved in about 10 min and would remain above this level for 5.8 h. The study found that intramuscular administration of TXA was well tolerated and rapidly absorbed, and UK paramedics are now authorized to give intramuscular TXA in the pre-hospital phase [22]. For example, the operational protocol of the West Midlands Ambulance Service in the UK states that 'If intravenous administration cannot be given quickly and the intramedullary route is not appropriate, the intramuscular route may be chosen with consideration given to administration to the larger muscle (or divided administration to the smaller muscle). For adults, 2 × (500 mg/5 mL) should be administered intramuscularly to separate sites." (West Midlands Ambulance Service operational protocol not published, personal communication from West Midlands Ambulance Service to Ian Roberts).

After intramuscular injection of TXA (which takes seconds), therapeutic TXA levels are achieved in about 10 min—almost certainly faster than with an intravenously injection, taking into consideration the time taken to cannulate and that TXA has to be injected over 10 min (the product characteristics overview recommends that TXA should be injected slowly intravenously at a rate of about 1 ml/min, so it takes at least 10 min to administer 10 ml) [23] More importantly, qualitative work shows that paramedics dislike the slow intravenously injection and often decide to leave it until the patient gets to the emergency department [24]. Other issues of concern could be addressed if TXA could be injected intramuscularly in pre-filled syringes, reducing the burden on emergency services, reducing the time spent at the scene and reducing the number of incident accidents.

## Conclusions

TXA has the potential to improve survival rates after trauma and has been widely used among many high-income populations, saving countless lives. However, there are still countries that are not using it to its fullest potential for the benefit of patients. New data on the use of TXA, including its use through the intramuscular route, in the pre-hospital setting justifies a re-evaluation of rules governing TXA use by emergency responders in many countries.

## Data Availability

The data for the CRASH-2 and Woman trials are available on the LSHTM data sharing website Freebird.

## Abbreviations

TXA: Tranexamic acid
TPA: Tissue plasminogen activator
ATACAS: Aspirin and Tranexamic acid for Coronary Artery Surgery
POISE-3: Perioperative Ischemic Evaluation-3
CRASH: Clinical Randomisation of an Antifibrinolytic in Significant Haemorrhage
RCT: Randomized controlled trial
TBI: Traumatic brain injury
WOMAN: World Maternal Antifibrinolytic
WHO: World Health Organization
